# The Over-the-Counter Medicines Market in Poland

**DOI:** 10.3390/ijerph192417022

**Published:** 2022-12-18

**Authors:** Dorota Lasota, Dagmara Mirowska-Guzel, Mariusz Goniewicz

**Affiliations:** 1 Department of Experimental and Clinical Pharmacology, Medical University of Warsaw, 02-097 Warsaw, Poland; 2 Department of Emergency Medicine, Medical University of Lublin, 20-081 Lublin, Poland

**Keywords:** over-the-counter drugs, OTC drugs, drug market, drug sales, pharmacy, side effects

## Abstract

The market of over-the-counter drugs, so-called OTC drugs, is a dynamically developing market driven primarily by self-medication. Their use does not require consultation with a physician, and the patients themselves decide to take them. The distribution of OTC medications in the pharmaceutical market in Poland is diversified. These drugs could be purchased at a pharmacy but also at a supermarket, gas station, or via the internet. The low involvement of public funds in spending on drugs, a relatively small percentage of pharmacy sales of reimbursable prescription drugs, and the difficult access to physicians or general consent to drug advertising all create ideal conditions for creating demand for these drugs. Among the European countries, Poland also has the largest share of OTC drugs in the entire pharmaceutical market, and the percentage of OTCs (without supplements) in the whole drug market in Poland continues to grow. Unfortunately, the non-pharmacy market for the sale of OTC drugs is not adequately controlled in practice, and Polish legal regulations regarding the sale of medicines outside pharmacies are among the most liberal in the European Union. However, this does not change the general attitude of consumers toward purchasing OTC drugs. In fact, further growth of the OTC drug market is forecast. Self-medication will undoubtedly play an important role in the trends which may shape this market in the coming years.

## 1. Introduction

The market for over-the-counter drugs has been developing dynamically. Every year, more and more new medicinal products available over the counter are registered. Their promotion accounts for the highest percentage of money spent on advertising. Although these drugs are considered safe, their incompetent use may pose the risk of numerous side effects, dangerous to the patient’s health and life.

Over-the-counter (OTC) is the official name for medications not requiring a prescription. Their use does not require consultation with a physician, and the patient decides to take them. They are used symptomatically, on an ad hoc basis, most often in the period preceding the visit to a physician, in conditions which are easy to diagnose by the patient, such as pain, colds, or gastrointestinal disorders.

In order for a medicinal product to be authorized as an OTC drug, it must be safe. The main problems with the use of OTC drugs are overdosing and the possibility of various interactions with other medications taken. Therefore, the patient may experience an increase in numerous ailments and complications, which contribute to the rise in the systemic costs of treatment. Unfortunately, most people taking over-the-counter medications are not fully aware of these threats.

## 2. Categories of Drug Availability in Poland

The criteria for the availability of over-the-counter medicinal products are defined by the World Health Organization (WHO) and in Europe by the European Medicines Agency (EMA).

The institution which admits medicinal products to the market in Poland is the office for registration of medicinal products, medical devices, and biocidal products (URPLWMiPB). These products are classified, among others, in the assigned availability category. The pharmaceutical law of 6 September 2001 distinguishes five categories of medicinal products’ availability. According to article 23a para. 1 of this act, these are the following products:

- Issued without a prescription (OTC, over-the-counter);
- Issued on a prescription (Rp);
- Issued on a prescription for restricted use (Rpz);
- Issued on a prescription - this group includes drugs or psychotropic substances, specified in separate regulations (Rpw);
- Used only for in-hospital treatments (Lz) [1].

Assigning a given product to one of the five categories is of practical importance as it determines the legal status of a medicinal product. It affects the possibility of re-founding a drug, the method of advertising it and labelling its outer packaging, as well as its availability in non-pharmacy trade. The criteria for classifying specific medicinal products to a specific availability category are regulated by the regulation of the minister of health of 14 November 2008 [2]. OTC drugs include products that do not threaten health and life when used correctly, do not contain narcotic drugs or psychotropic substances, or do not cause a risk of addiction or special supervision during their use. These drugs are generally low in toxicity, lack mutagenic and carcinogenic properties, and have a relatively low risk of serious side effects and interactions.

In some cases, however, the category of drug availability may be changed, which is called a switch, e.g., when the side effects caused by the drug outweigh the benefits resulting from the treatment, the category is changed availability from OTC to Rp (the so-called reverse switch). Another reason may be that a given medicinal product has been used repeatedly against the indications [3]. Changes in the category of availability of medicinal preparations from Rp to OTC are sometimes controversial. In recent years, the most doubts were raised regarding the OTC sale of medicaments containing ketoprofen, sildenafil, or nasally administered mometasone [4].

## 3. OTC Medicine Sales

The distribution of OTC drugs in the pharmaceutical market in Poland is diversified. These products could be purchased at a pharmacy but also at a supermarket, gas station, or via the internet.

Pharmaceutical care and patient health safety are the advantages of selling through pharmacies. According to the PMR market experts (PMR) report, stationary pharmacies are the main place to buy OTC drugs in Poland. Approximately 90% of consumers choose them [5]. Pharmacies may also conduct mail-order sales of over-the-counter medications after meeting the conditions specified for this form of activity and reporting such an intention to the competent voivodeship pharmaceutical inspector [6,7,8].

A patient driven by convenience praises the possibility of purchasing an OTC drug in a supermarket or gas station [6,9]. Non-pharmacy turnover accounts for approximately 5% of the market for over-the-counter medicinal products [10].

The running of pharmacies and the sale of over-the-counter drugs outside of pharmacies varies across Europe. Thus, in twelve European Union (EU) countries, non-pharmacy trade in drugs does not exist, and pharmacies have a monopoly on selling medicinal products. They are Belgium, Cyprus, Estonia, Finland, France, Greece, Lithuania, Luxembourg, Latvia, Malta, Slovakia, and Spain; however, in Finland, non-pharmacy marketing of nicotine replacement therapy products is allowed. In six EU countries, non-pharmacy turnover is allowed for a narrow group of drugs. These are Austria, Bulgaria, Croatia, Germany, Portugal, and Romania. In Austria, non-pharmacy sales mainly concern medicinal products of plant origin. In nine EU member states, non-pharmacy trade is authorized. The ministries of health in these countries define which drugs may be sold at points of sale other than pharmacies. These are the Czech Republic, Denmark, the Netherlands, Ireland, Poland, Slovenia, Sweden, Hungary, and Italy. Each of the aforementioned countries (except Poland) has additional requirements and restrictions regarding non-pharmacy trade [10].

In Poland, retail trade for over-the-counter medicinal products may be conducted by non-pharmacy outlets such as herbal and medical stores, specialist medical supply stores, and generally accessible stores [1]. The pharmaceutical law obliges the minister of health to define a list of medicinal products admitted to non-pharmacy trade, qualifications of persons dispensing medicinal products under such trade, as well as requirements that commercial establishments should meet to ensure the safety of use, storage, and distribution of drugs. Currently, the rules and scope of non-pharmacy trade in medicinal products are regulated by the bill of the minister of health of 21 December 2021, on the list of medicinal products that may be admitted to trading in non-pharmacy outlets and pharmacy outlets, and the criteria for classifying these products to individual lists [11].

Online pharmacies also record very high sales of OTC drugs in Poland, especially after the legal changes in 2018, when online sales of OTC drugs became legal in all central European countries. The exception was Slovakia, where OTC drugs reimbursed under the obligatory health insurance premium were excluded from online distribution [12]. Two main reasons for the great interest in this form of purchasing OTC drugs are the growing percentage of Poles using the internet, including the elderly and those living in smaller towns, and the lower price.

The dynamically growing segment of mobile commerce (m-commerce), i.e., the area of e-commerce in which mobile devices (mobile phones) play an essential role, also contributes to the development of the pharmaceutical market [9].

## 4. The Most Frequently Sold OTC Drugs

The low involvement of public financing in refunding medications, a relatively small percentage of pharmacy sales concerning reimbursable prescription drugs, problematic access to physicians, and general consent to drug advertising, have created ideal conditions for creating demand for OTC drugs. These excessive OTC purchases lead to an increase in self-medication with over-the-counter medications.

**Figure 1:**
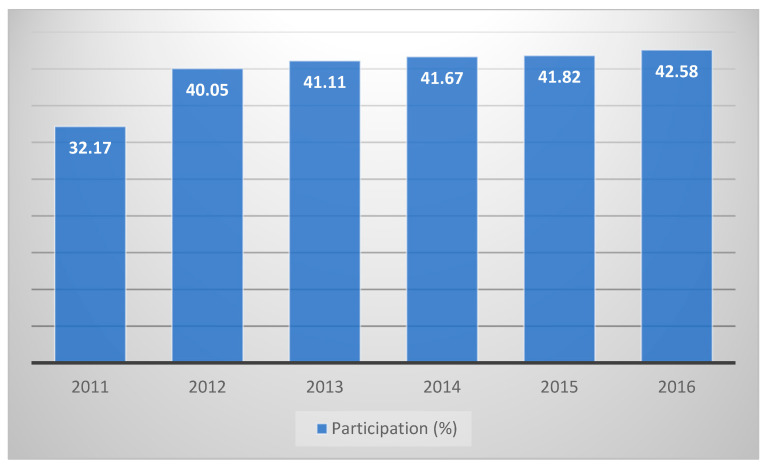
Share (%) of OTC preparations (without dietary supplements) in the drug market in Poland, 2011–2016. Source: own study based on [10].

Poland is at the forefront of European countries regarding the cost of self-treatment per capita. Also, considering the fact that drugs in Poland are among the cheapest in Europe, Poland stands at the forefront in terms of their consumption. According to the report of the association of pharmacists employers of polish pharmacies (ZAPPA), statistically, a Pole spends 181 dollars per year on OTC drugs and dietary supplements [13], and according to the data of the association of the European self-care industry (AESGP), the sale of products used in self-treatment exhibits a growing tendency in Poland. Poland also has the largest share of OTC drugs in the entire pharmaceutical market among the European countries. The share of OTC products (excluding supplements) in the whole drug market in Poland continues to grow (Figure 1).

It is worth mentioning the essential difference between an OTC drug and a supplement, which are the properties of these products. Supplements do not cure disease; they are, by definition, concentrated sources of nutrients, vitamins, and minerals. Their task is to enrich our diet with ingredients not supplied to the body in appropriate amounts with food. A significant difference is also in the registration process of both products.

According to Pharma Expert, in the OTC drug market, most of the turnover is accumulated under one category, i.e., analgesics [10,14,15,16]. Since Poles “consume” approximately 2 billion analgesics annually, with as many as 6% taking them prophylactically, i.e., before the pain occurs, and 40% even with minor pain symptoms. The excessive consumption of painkillers is, among others, a result of the unlimited availability of these drugs. Their excessive consumption also results from the widespread advertising of OTC drugs, which is a tool supporting their sale and their producers, chain pharmacies, and generating income for the media [10].

Generally, accessible stores are responsible for the sale of 45% of painkillers in Poland. Ibuprofen and paracetamol are among the most popular products in this category. The value of their sales in the non-pharmacy channel accounts for over 47% of sales in the Polish market. The analysis of the number of purchase points confirms the perception of generally available stores as the primary source of supplying Polish consumers with painkillers. The pharmacy distribution channel currently accounts for approximately 15 thousand pharmacies, while the non-pharmacy market creates approximately 105 thousand retail outlets, of which approximately 90% distribute OTC drugs. In an average hypermarket in Poland, you could find approximately 109 products with the status of a drug, dietary supplement, or herb [10].

**Figure 2:**
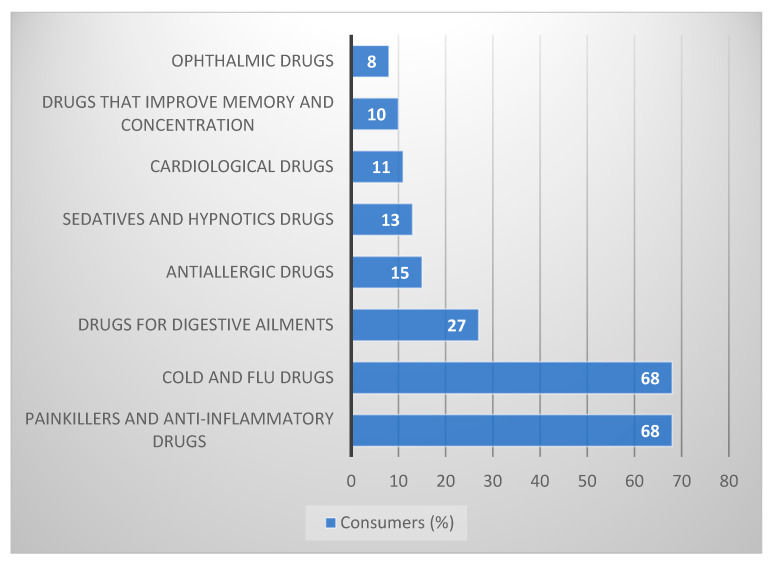
Consumers (%) using OTC drugs. Source: own study based on [10].

The next group in the ranking of the most commonly used OTC medications are products for influenza, colds, and flu-like infections (Figure 2). The season of such infections usually lasts from November to March, but the number of cases varies yearly. In record years of disease (2002, 2003, and 2009), Poles bought over 75 million packages of OTC drugs and dietary supplements. In 2015, Poles bought over 50 million products for the diseases mentioned above, spending almost PLN 700 million on them [17]. PEX PharmaSequence data shows that only from June to November 2020, Poles bought over 80 million of these pharmaceuticals (both drugs and dietary supplements) [18].

## 5. Health Risks Associated with the Use of OTC Medicines

The number of generally accessible stores where it is possible to purchase over-the-counter medicines in Poland is so large that there is no guarantee of exercising real control over both the storage and dispensing of medicinal products. In practice, the non-pharmacy market for the sale of OTC drugs is not controlled by the state pharmaceutical inspection, and Polish legal regulations regarding the sale of medicines outside pharmacies are among the most liberal in the European Union. The supreme audit office (NIK) report published in March 2016 on the control of the activities of the state pharmaceutical inspection bodies in 2012–2015 showed that they ineffectively supervised the trade in medicinal products. The recommendations of the supreme audit office in the report also indicated that it is necessary to intensify the supervision of non-pharmacy drugs and to verify by the minister of health the list of medicinal products admitted to sale outside pharmacies [19].

The association for drugs only from the pharmacy, has issued a comprehensive report, “Non-pharmacy trade in OTC drugs - safety, economy and patient expectations.” According to this report, the non-pharmacy market is out of control, and the state incurs very high medical costs related to the inappropriate use of OTC drugs. The report also reveals a significant increase in the number of preparations available in the non-pharmacy market. In 2009, 525 preparations were available on this market, and in 2017 this number was estimated at 3000 [10].

Over-the-counter drugs from a generally accessible pharmacy are dispensed by a pharmacist or pharmaceutical technician as part of their professional qualifications. The pharmacist is a key factor in selecting decisions concerning self-treatment among patients. The pharmacist is often the first person to whom a patient with a health problem turns when contact with a doctor is problematic. Pharmaceutical advice is a practical professional activity of a pharmacist, which aims to provide an interested customer in a pharmacy with assistance and advice on the prevention of diseases and the use of the most appropriate over-the-counter pharmaceuticals in self-treatment. Based on their qualifications, knowledge, experience, and skills, the pharmacist may recommend the patient to seek medical advice immediately, warn against the use of a specific drug, indicate another more appropriate one, discourage the patient from taking the OTC drug, or indicate another method of treatment. According to the code of ethics of the pharmacist of the republic of Poland, a pharmacist is obliged to provide professional, equally caring assistance to all persons who require support and to provide necessary advice in the selection of medications that do not require a medical prescription [20].

Unfortunately, patients who purchase OTC drugs outside pharmacies have limited access to information on their proper use and storage. The requirements for the qualification of persons dispensing medicinal products in non-pharmacy trade, defined in the regulation of the minister of health of February 2009 on the qualification of persons distributing medicinal products in non-pharmacy outlets, as well as the requirements to be met by the premises and equipment of these establishments and pharmacy outlets, are very general [21]. They detail the need to have knowledge concerning the use and storage of the drugs sold based on the information contained in the package leaflets of the products. However, the sellers in the non-pharmacy channel do not have specialist knowledge and do not provide pharmaceutical care to their customers. This lack of knowledge of OTC sellers causes the patient to be exposed to the wrong drug and the multiplication of doses of the active substance used. There are often reports in the media about, for example, overdosing on popular painkillers. The problem of producing intoxicants based on OTC drugs is also publicized [10].

Fortunately, for many patients, the primary source of information on combining various drugs, and potential interactions with other medicinal products and food, are physicians and pharmacists. According to the body of literature, 55% of respondents asked about the possibility of combining pharmaceuticals, 55% read the leaflet attached to the drug packaging, and 34% checked the most important information in it. In addition to physicians and pharmacists, the leaflet is one of the main sources of information on drug interactions (54%) and the combination of pharmaceuticals with other foods or herbs (42%). Nevertheless, the internet is the source of information on medicines for an equally large number of respondents. A total of 20% of respondents obtained knowledge about combining different drugs from the internet, while information about which products should not be combined with specific pharmaceuticals was performed by 18% of respondents [7].

Meanwhile, the misuse of OTC drugs also poses a serious threat to public health. Adverse drug reactions deteriorate the patient quality of life, as well as increase both the number of hospitalizations and deaths. In many cases, long treatment is necessary, which creates an additional cost for the health care system. The importance of this problem is evidenced by the figures, which demonstrate that drug-related complications impact 10.8% to 38% of patients. The data from the US and Sweden show that drug-related complications rank among the top ten causes of death, causing 100,000 deaths per year in the US and expenses of approximately 3 billion dollars. In European Union (EU) countries, 20% of the financial resources allocated to health care are related to the diagnosis and therapy of adverse drug reactions [22,23,24]. According to WHO estimates, approximately 13% of hospitalizations are the result of improper medication. The top five causes of hospital admissions include serious complications related to the use of these drugs [25]. This is especially true for over-the-counter drugs from the group of Non-steroidal anti-inflammatory drugs (NSAIDs) [13], which are responsible for approximately 25% of all drug-related complications [10,26,27,28].

In Poland, the treatment of side effects, regardless of whether they relate to reimbursed drugs, over-the-counter drugs, or even dietary supplements, means huge expenses covered by the national health fund (NFZ). The total annual cost of hospital treatment in Poland, financed by the national health fund, is over PLN 42 billion. The adoption of WHO estimates would mean that PLN 5.2 billion is the public cost of treating cases related to inappropriate drug intake in this country [13]. The costs of public health related to the treatment of the improper use of drugs purchased in the non-pharmacy trade are estimated at a minimum of PLN 130 million per year [10].

In mid-2017, the ministry of health withdrew from attempts to introduce changes aimed at limiting the availability and reducing, above all, the doses and packaging of OTC drugs. The ministry justified these steps using economic reasons, i.e., the interests of producers and retail outlets. In light of the report of association for drus only from the pharmacy “Non-Pharmaceutical Trade with OTC Drugs—Safety, Economy and Patient Expectations”, this argument became false, as the share of drugs in the turnover of an average retail outlet was below 1% [10]. The regulations governing the non-pharmacy drug trade require changes, first of all, for the sake of patient health and safety and the interest of the state. This market should be organized and adapted to the solutions used in Europe. The governments of most European countries are aware of the negative social impacts of the excessive expansion of the non-pharmacy market and are trying to regulate it. The reason is the alarmingly growing number of poisonings related to paracetamol use, among others [14,29,30]. Poland is negatively distinguished by, among other things, no limitation in the number of drug packages or doses sold outside pharmacies, especially non-steroidal anti-inflammatory drugs and paracetamol [7,12,28,29,31]. Requirements for the composition, pharmaceutical form, strength, and content of the active substances in the packaging of a medicinal product or the packaging size of a medicinal product for medicinal products that may be authorized for non-pharmacy marketing are specified in annexes 2 and 3 to the regulation of the minister of health of 21 December 2021 on the list of medicinal products which may be admitted to trading in non-pharmacy outlets and pharmacy outlets, and the criteria for classifying these products to individual lists [10,11].

Moreover, the non-pharmacy market in Poland is also practically unprotected against the introduction of counterfeit drugs. Although the so-called fake amendment, which adjusts Polish law to the EU system preventing the sale of counterfeit medicines, imposed several obligations on pharmacies to protect against the placement on the market of drugs of unknown origin (special codes and readers for reading them, online batch control, etc.). These laws, however, only work in pharmacies.

According to WHO data, even 1% of drugs marketed in developed countries may be counterfeit. Falsified medicines available on the internet account for approximately 50% of the products offered. Globally, the number of counterfeit drugs may account for approximately 10% of the global drug market, and in some developing countries, it may account for more than 30% of commercially available drugs [32]. According to EU experts, the annual costs of purchasing counterfeit medicines by consumers across Europe amount to approximately EUR 850 million, with the size of the Polish market for such goods estimated at EUR 62 million by the European commission. The WHO estimates that the global market for counterfeit drugs is worth 200 billion dollars a year. According to the WHO, one package of counterfeit medicine in Poland may be sold in one hundred packages [10].

## 6. Forecasted Developmental Directions for the OTC Market in Poland

**Figure 3:**
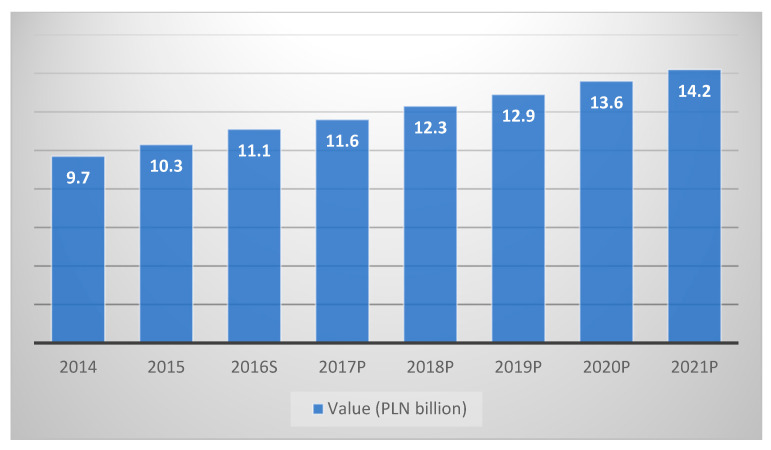
Value of the OTC market in Poland, 2014–2021. P—prognosis, S—estimation. Source: own study based on [35].

The OTC market and its development are strongly correlated with the economic situation in Poland, especially with the status of households. Good macroeconomic indicators have been the driving force behind the growth in this segment for many years (Figure 3). Undoubtedly, among the trends which may shape this market in the coming years, self-medication plays an important role, driving the increase in sales of OTC drugs and changes in health policy, which may lead to changes in the method of distributing funds from the health care sector and access to medicines, as well as demographic factors, i.e., a growing number of older adults generating significant demand for both drugs and dietary supplements. According to the PRM market experts report “OTC products market in Poland 2021. Market analysis and development forecasts for 2021–2026”, sales in the OTC segment will grow by 4% to 6% annually in the coming years [33,34,35].

## 7. Conclusions

The greater availability of over-the-counter medicines renders it easier to reach for products assisting in relieving ailments such as pain or infections of the upper respiratory tract. The percentage of Poles who limit physician visits while undertaking self-treatment is alarmingly high. This upsurge is confirmed by the quantitative increase in sales of pharmaceuticals in categories such as analgesics, flu and cold medications, and vitamin preparations. Self-medication is predicted to play an essential role in the trends shaping the OTC market in the coming years, driving sales growth for these products.

However, the universality of self-treatment carries many threats that require careful legislation shaping and the fulfilment of control functions by state authorities. Many issues require clarifications, or even the introduction of new solutions, adjusted to the changes in medical knowledge, education level, and social awareness.

The individual responsible for the responsible sale of OTC drugs and pharmacotherapies should be adequately educated and prepared for this task, guaranteeing the proper performance of their duties. A pharmacist is such a person as a representative of a medical profession of public trust who guarantees the appropriate realization of their social tasks following the principles of ethics and professional deontology. Unfortunately, the role of pharmacists in healthcare in Poland is significant but underestimated.

## Data Availability

Not applicable.
